# Neurophysiological Correlates of Cognition as Revealed by Virtual Reality: Delving the Brain with a Synergistic Approach

**DOI:** 10.3390/brainsci11010051

**Published:** 2021-01-05

**Authors:** Sachin Mishra, Ajay Kumar, Parasuraman Padmanabhan, Balázs Gulyás

**Affiliations:** 1 Cognitive Neuroimaging Centre, 59 Nanyang Drive, Nanyang Technological University, Singapore 636921, Singapore; sachin.mishra@ntu.edu.sg (S.M.); ajaynsysu@mem.nsysu.edu.tw (A.K.); 2 Institute of Biomedical Sciences, National Sun Yat-sen University, Gushan District, Kaohsiung 804, Taiwan; 3 Department of Mechanical and Electro-Mechanical Engineering, National Sun Yat-sen University, Gushan District, Kaohsiung 804, Taiwan; 4 Department of Clinical Neuroscience, Karolinska Institute, 17176 Stockholm, Sweden; 5 Lee Kong Chian School of Medicine, Nanyang Technological University, Singapore 608232, Singapore

**Keywords:** virtual reality, neuroimaging, neuromodulation, perception, stimulation

## Abstract

The synergy of perceptual psychology, technology, and neuroscience can be used to comprehend how virtual reality affects cognition of human brain. Numerous studies have used neuroimaging modalities to assess the cognitive state and response of the brain with various external stimulations. The virtual reality-based devices are well known to incur visual, auditory, and haptic induced perceptions. Neurophysiological recordings together with virtual stimulations can assist in correlating humans’ physiological perception with response in the environment designed virtually. The effective combination of these two has been utilized to study human behavior, spatial navigation performance, and spatial presence, to name a few. Moreover, virtual reality-based devices can be evaluated for the neurophysiological correlates of cognition through neurophysiological recordings. Challenges exist in the integration of real-time neuronal signals with virtual reality-based devices, and enhancing the experience together with real-time feedback and control through neuronal signals. This article provides an overview of neurophysiological correlates of cognition as revealed by virtual reality experience, together with a description of perception and virtual reality-based neuromodulation, various applications, and existing challenges in this field of research.

## 1. Introduction

Perception is defined as the organization, recognition, and interpretation of sensory information to characterize and understand the presented information or the environment. Interaction between perceptual psychology, graphics technology, and brain sciences can be used to understand what virtual reality (VR) does to our brains. The VR-based devices are well known to incur dynamic audio–visually induced perceptions in humans [1].

To study the cognitive state of a person is of crucial importance in the fields of human factors and ergonomics. A reliable measurement of the cognitive state is known to assist in the treatment of various neuronal disorders and could also allow for enhancements in human–machine interface designs and brain–computer interfacing (BCI) [2,3]. At present, numerous studies have used neuroimaging modalities and their hybrid versions [4] to assess the cognitive state, reporting the cognitive functions and responses to be associated with event-related potentials, functional responses, and fluctuations of different frequency bands of brain oscillations.

There are various neurophysiological data recording tools, such as electroencephalography (EEG), magnetoencephalography (MEG), and magnetic resonance imaging (MRI) that are well known to record resting state and stimulated state brainwave activities, which can be further quantified in different frequency bands [2]. Electrophysiological approaches can be used as a tool to study neuro-dynamics with high temporal resolution in the order of milliseconds, and might be used as an ensemble of biomarkers that is significantly required in the assessment of cognitive changes observed in various activities and challenge conditions. Significant results from experimental approaches can aid in the interpretation of the reason for the transition of a cognitive state, and tracing of the neurophysiology behind the various activities, including VR.

Researchers are working with the approaches of BCI in the field of biomedical, education, engineering, marketing, healthcare, and gaming by blending human physiological data, such as EEG with systems of VR [1,5,6,7]. These brain responses combined with VR, enable in correlating humans’ physiological signals with their tasks in the designed environment. Thus, the effective combination of the two has been used to study users’ cognitive states, assess and train them in navigation, investigate their perceptions, and various other dimensions.

There are various benefits of VR-based experiments over conventional experimental instruments, such as dimensional control of the stimulus environment, providing a more realistic protocol for the presentation and reaction of stimuli. It is known to incur visual, auditory, and haptic induced perceptions with greater control and possibilities, and can modulate human perception and motor performance. Simulations with VR systems provide an enhanced and immersive experience of a sense of presence and allow for skill improvement during VR training [8]. These systems helped researchers to study the contributions of movement-versus-outcome-related sensory feedback for the sense of agency [9]. The study of body ownership is another field where VR systems can be incorporated with neuroimaging and utilized in the development of neuroprosthetics. Illusory perception of virtual or missing body parts triggers motor evoked potentials and corticospinal pathways, which are of great use in the study of body ownership [10]. Visual representation of movement is another field that can be stimulated through VR systems and the triggered neurophysiological responses in terms of event-related potentials can assist to characterize the neural signature of motor imagery tasks [11]. Another area is the study of sensorimotor integration, whereby its enhancement assists in post-stroke rehabilitation, and VR systems with multisensory stimulation help in gait rehabilitation [12].

This review discusses various approaches and applications of VR integrated neuroimaging, and provides an overview of neurophysiological correlates of VR. The article provides insights into the efficacy of VR in diverse fields, such as neurorehabilitation and therapy, neuromodulation, cognitive response, perception, sense of presence, spatial navigation, memory encoding and retrieval, BCI and its feedback control, and neuromarketing.

## 2. The Synergy of Virtual Reality and Neuroimaging

The mapping of neuronal activity using neurophysiological recordings together with virtual reality devices is known to be a significant approach to study the alteration of the psychobiological state of mind during VR induced dynamic perceptions. Together with VR devices, these brain scanning modalities can be used to get comparative data of brain response (such as power spectrum density values together with corresponding topo maps, event-related potential, and responses) in pre-activity, activity, and post-activity sessions of VR-based cognitive tasks. This is known to show significant alterations in various frequency bands of neuronal oscillations [13,14]. The oscillatory signals from our brain provide us with an impactful picture of the mental state of the person. Thus, they could be used to study the cognitive state during perception. These neural oscillations, also known as brain waves, have the potential to indicate the state of mind under various stimulated and resting-state conditions. Furthermore, research is ongoing to focus on decoding brain activity in various domains of cognition with augmented reality experience, which provides an enhanced level of interaction with the surrounding environment [15,16,17].

Traditionally, neuronal oscillations can be clustered based on the frequency bands, namely, Delta wave (<4 Hz), indicating slow-wave sleep, Theta wave (4–8 Hz), occurring during drowsiness, Alpha wave (8–12 Hz), indicating an awakened state, Beta wave (13–30 Hz), arising only during the alert state, and Gamma wave (30–60 Hz), designating a hyperactive state [18]. These approaches can be used as a tool to study neuro-dynamics with high temporal resolution in the order of milliseconds and might be used as an ensemble of biomarkers that is significantly required in the assessment of cognitive changes observed during the use of VR-based devices.

Various neuroimaging modalities are explored by researchers in studying cognition with the VR experience. For example, EEG was used by researchers to study the responses evoked by the presentation of specific three-dimensional (3D) virtual tunnels with navigational images [19], and to characterize the neural generators of the brain oscillations in a virtual tennis court, related to motor imagery [20]. Moreover, Roberts et al. [21], for the first time, combined an optically pumped magnetometer (OPM) based MEG system with a VR system and recorded alpha oscillations and visual evoked fields in the presence of VR stimulations. In addition, functional imaging techniques, such as functional MRI (fMRI), are also incorporated in VR stimulations [22,23,24].

**Figure 1:**
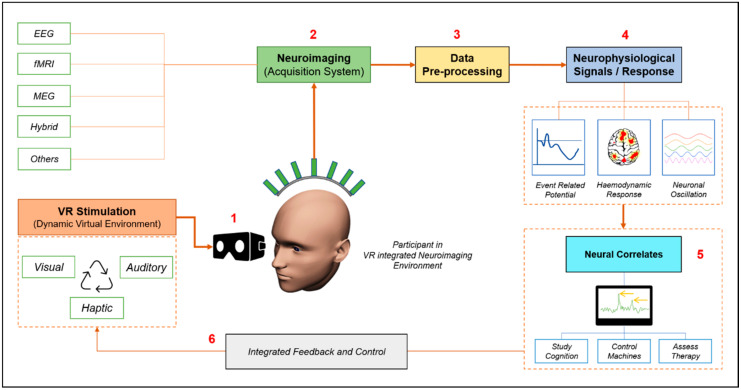
A brief layout of this virtual reality (VR) integrated neuroimaging approach.

Although it is crucial to understand the engineering and technological aspects of VR, neuroscientists and neurotechnologists are keen on comprehending the synergistic integration of VR with neuroimaging. A brief layout of this integrated approach is shown in Figure 1. Various developed, customizable, and ready-made VR-based setups can be used for research. These VR devices can be a head-mounted or screen-based system. As shown in the figure, the participant is expected to wear the VR device and the neuroimaging modality is attached. The virtual environment created by the system works as a customizable stimulus that is dynamic in audio, visual scenes, and occasionally in haptics. The experiment constitutes a recording of data during the stimulation, following which, it will either be processed to visualize the neural correlates, or directly integrated with the machine, such as the BCI system. The setup provides immense possibilities for studying the cognitive state and neuromodulation.

## 3. Neurophysiological Correlates of Cognition as Revealed by Virtual Reality

**Figure 2:**
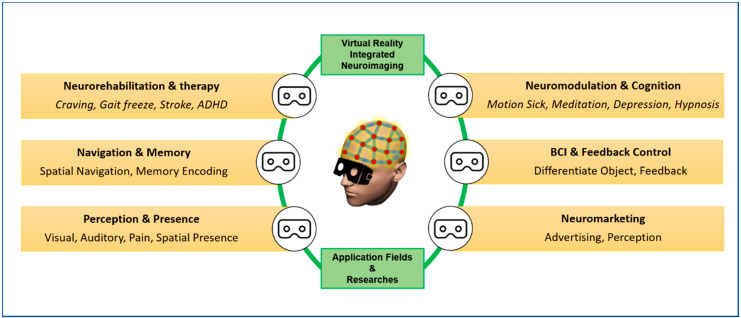
A brief overview of the applicability of VR integrated neuroimaging.

The study of neural signals and functional response of the brain through various neuroimaging modalities such as EEG, MEG, MRI, etc., integrated with VR environment-based stimulation, have immense capabilities to delve into human perception, memory, cognitive state, dysfunctions, behavior, and other aspects. Various researchers have successfully tried to correlate the neural response in the virtually designed environment. Various approaches and applicability of this synergistic approach are described in this section. A brief overview of the applicability is shown in Figure 2.

### 3.1. Neurorehabilitation, Neuroprosthetics, and Cognitive Behavioral Therapy

The study of the neural correlates of VR for therapeutic applications is an evolving field and can assist to treat various dysfunctions. Lee et al. [25] utilized VR-based therapy with multisensory stimulation to reduce the craving for alcohol. The therapy consisted of a series of scenes associated with virtual alcohol cues, relaxation, and aversive stimulation. The VR goggle-based experiment, which included the selection of a type of alcoholic beverage and drinking situation to view during the high-risk situation, together with EEG recording from 19 monopolar electrode sites, showed an increase in alpha activity in the frontal region after the 10th session, together with a decrease in craving for alcohol in alcohol-dependent patients. Customized VR scenes with multisensory modes of stimulation showed improved therapeutic efficacy than traditional cognitive therapy. Similarly, Moon et al. [26] worked with an fMRI-based experiment in a virtual environment with cue exposure treatment to reduce the craving for nicotine.

**Figure 3:**
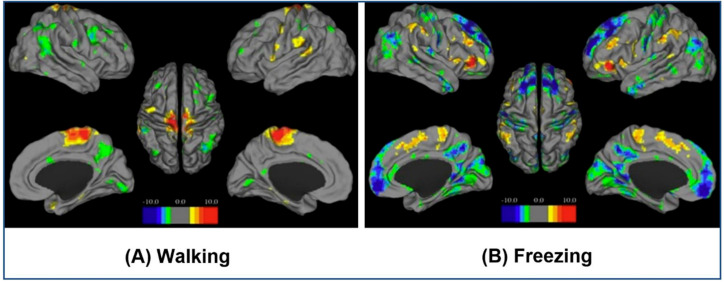
Distinct Blood-oxygen-level dependent (BOLD) patterns observed during walking and episodes of freezing with VR based method. (**A**) Whole brain statistical parametric mapping (SPM) maps showing cortical activation pattern (represented as contrast values from SPM analysis) comparing ‘‘walking’’ with observing showing bilateral activation of sensorimotor regions known to mediate movement of the lower limbs (statistical map threshold significant at *p* < 0.001; false discovery rate corrected to *p* < 0.05). (**B**) Whole brain statistical parametric mapping (SPM) maps showing pattern of cortical activation and deactivation (represented as contrast values from SPM analysis) present during freezing episodes (*p* < 0.001; false discovery rate corrected to *p* < 0.05). Periods of freezing demonstrated marked bilateral activation in the pre-supplementary areas, the motor cortices, dorsolateral and ventrolateral prefrontal cortices, as well as posterior parietal regions. In contrast, there was marked bilateral deactivation of the frontopolar cortices and the precuneus. Upper left corner: Right hemisphere lateral, upper right corner: Left hemisphere lateral, lower left corner: Left hemisphere medial, lower right corner: Right hemisphere medial, and center: Both hemispheres dorsal. Adapted from the *Journal of Clinical Neuroscience* Shine, J.M., Ward, P.B., Naismith, S.L., Pearson, M., Lewis, S.J. (2011). Utilizing functional magnetic resonance imaging (fMRI) to explore the freezing phenomenon in Parkinson’s disease. 18(6), 807–810. Copyright © 2020 Elsevier Ltd. All rights reserved, with permission from Elsevier. Reference [27].

Functional neuroimaging using fMRI together with VR can be used to reveal the underlying neural correlates of motor functions in neurodegenerative diseases, such as freezing behavior in Parkinson’s disease (PD). Sine et al. [27] demonstrated a real-time VR-based walking task, both, with and without dopaminergic medication observed with fMRI. Freezing behavior was provoked by this VR-based stimulation method and distinct Blood-oxygen-level dependent (BOLD) patterns were observed during walking and episodes of freezing. The distinct pattern of activation and deactivation underlying the freezing sensation is shown in Figure 3.

**Figure 4:**
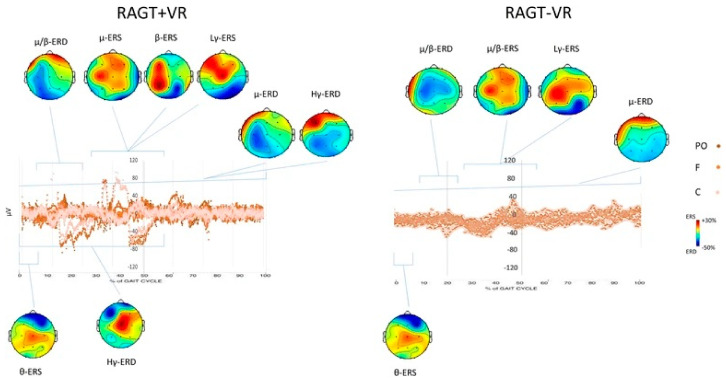
Average changes at second day of the first week of treatment (T_POST_) as compared to last day of the last week of treatment (T_PRE_) in scalp Event-Related Potential (ERP) projections relatively to the full gait cycle. The left and right hemispheres plots correspond to the affected and unaffected ones, respectively. Event-related synchronization (ERS) and Event-related de-synchronization (ERD) are masked in red and blue tones, respectively, whereas nonsignificant differences are in green. All rights reserved, with permission from Springer Nature. Reference [28].

It is observed that VR-based setups can be used in neurorehabilitation by inducing motor function recovery, especially in stroke patients. EEG oscillations can be used to analyze the neurophysiological correlates of motor function recovery induced by robotic-assisted gait training device together with VR scenes, as described by Calabro et al. [28]. It was observed that high-γ and β bands had stronger event-related spectral perturbations together with large frontocentral cortical activations in the affected hemisphere (shown in Figure 4). It was demonstrated that the robotics-based rehabilitation system combined with VR entrained several brain areas and stronger cortical activations were observed, due to VR feedback, in the fronto-parieto-occipital areas, probably referring to the mirror neuro system. August et al. [29] demonstrated the possibility of activating primary and secondary motor areas through VR-based hand exercise, which was useful for stroke patients. Similarly, Steinisch et al. [30] worked on neuro motor rehabilitation of the upper limb in stroke patients. They tried to integrate robotics, VR system, and EEG recording to train the participants with distinct movement patterns of the upper limb, through a VR training application. They used a passive robotic device for kinematic tracking together with compensation of gravity. The proposed system was demonstrated as a potential rehabilitation system for monitoring neuro-motor recovery.

Goncalves et al. [31] experimented with upper limb stroke patients using VR therapy with a Computed tomography (CT) scan, as an interactive intervention to induce neuroplasticity. Pre-and post-VR therapy sessions were compared and to study the relationship between the lesion and upper limb function, a voxel-based lesion-symptom mapping approach was used. It was observed that the functionality and ability of the upper limb improved after the therapy.

Merians et al. [32] developed a hand and arm training system with interactive virtual environments for stroke patients, which can assist in the activation of brain areas through visual feedback. They predicted using fMRI results that the visual augmentation can assist to facilitate functional neuroplasticity. Prochnow et al. [33] worked with a rehabilitation gaming system with stroke patients and demonstrated through fMRI data that recovery is possible through object processing, attention, and mirror neuron system involvement. The fMRI results showed that virtual target catching can activate frontal, parietal, temporal, cingulate, and cerebellar regions. Similarly, Xiao et al. [34] performed a preliminary study with subacute stroke survivors using a virtual reality-enhanced treadmill. The fMRI results for ankle dorsiflexion activity showed increased activation in the primary sensorimotor cortex of the lesioned hemisphere and supplementary motor areas of both sides for the paretic foot, after the intervention. This led to improved walking and cortical activation.

Various experiments helpful in neuroprosthetics and motor control and coordination activity can be also performed with the use of VR systems. For example, Bach et al. [35] developed and experimented with an MRI-compatible VR-based setup that can trigger illusory sensations with visual and tactile stimulations. The experiment demonstrated the fMRI response acquisition with illusory ownership experiences for a virtual limb. A highly susceptible subject was noted to show experience of ownership accompanied by the bilateral activity of ventral premotor cortex, secondary somatosensory cortex, extrastriate visual cortex, as well as right-hemispheric activation of the cerebellum. Menon et al. [36] developed an MRI-compatible system that can stimulate haptic interaction in an immersive virtual world. The device is useful in studying the correlates of neuromuscular control experiments involving interactive motor tasks, tactile perception, and visuomotor integration activities.

Moreover, VR stimulated neuroimaging can be utilized for cognitive behavioral therapy. Attention enhancement is one field where this synergistic approach is increasingly experimented on. Cho et al. [37] developed a VR-based attention enhancement system, integrated with EEG biofeedback. The study was based on EEG biofeedback treatment for Attention deficit hyperactivity disorder (ADHD), using Beta wave as feedback control, and showed significant results in attention enhancement.

### 3.2. Neuromodulation and Cognitive State

Neuromodulation through activities, such as meditation, is also possible through VR based devices. Choo et al. [38] used a gamification approach to develop software that can provide immersive virtual environments with guided meditation tracks for mindfulness activities, and supports EEG data acquisition to study the mental state of the person. They used a point system, “Serenity”, in the virtual system as a reward for mindfully meditating with the software and maintaining user interest in meditation. The software contained mindfulness scenes of realistically rendered and animated locales, such as Angkor Wat, with different meditation lessons. Emotiv EEG headset was used, which directly tracks the meditation score as well as other affective states. However, the experiment did not discuss the EEG data.

Driver cognitive response is also an area where researchers are experimenting with VR-based dynamic environment, together with neuroimaging, to study accidents. Lin et al. [39] worked with kinetic audiovisual stimuli delivering a VR device that can perform traffic-light motion simulation. The EEG signals were recorded and through Independent component analysis (ICA), noise-free Event-Related Potential (ERP) signals were obtained, which were further processed with a self-constructing neural fuzzy inference network, recognizing different brain potentials corresponding to red/green/yellow traffic events, and describing the cognitive state and response to task events. It showed that multiple streams of ERP signals represent operators’ cognitive states and responses to task events.

Silva et al. [40] experimented to study the response of the brain with changes in motion speed of a non-immersive 3D virtual stimulus. The visual-time reproduction task was varied at three-speed conditions and EEG data analysis showing theta band power in the dorsolateral prefrontal cortex, was observed. In all three-speed conditions, increased EEG theta power in the right dorsolateral prefrontal cortex, was observed, which was related to the accumulation of temporal pulses, further relating to attention and memory.

### 3.3. Perception

Perception is defined as the process of organization, identification, and interpretation of sensory information to perceive the environment. Visual perception is known to involve the sensorimotor system of the brain and VR-based setups can modulate neural oscillations and reveal the underlying process. Neural oscillation, which can be monitored with neurophysiological recordings, can assist to understand the correlates of perception.

Visual space is known to accompany a series of perceptual thresholds with functional sub-divisions and thus linked to behavior. Neuroimaging studies expose that visual perception of manipulable objects is related to motor capacities. Wamain et al. [14] studied the process of motor coding of visual objects in peripersonal space. The centro-parietal region was studied while perceptually judging the intrinsic or extrinsic properties of visual objects. The desynchronization of Mu rhythm was observed 300 ms after the object was presented. The results indicated that motor coding was dependent on the goal of the perceptual task.

The neural correlates of auditory distance perception are of interest to researchers. The spatial attributes are known to be correlated with the recognition of acoustic patterns. Mathiak et al. [41] tried to understand the neural mechanisms of auditory distance perception using MEG signals as variations in amplitude over both supratemporal planes. Hemispheric lateralization was indicated by enhanced pre-attentive responses over the right temporal lobe. It shows that the pre-attentive neuronal basis of amplitude discrimination is selective to spatial encoding. This helped to understand the temporal multisensory matrix that promotes the integration of spatial information across visual and auditory modalities.

**Figure 5:**
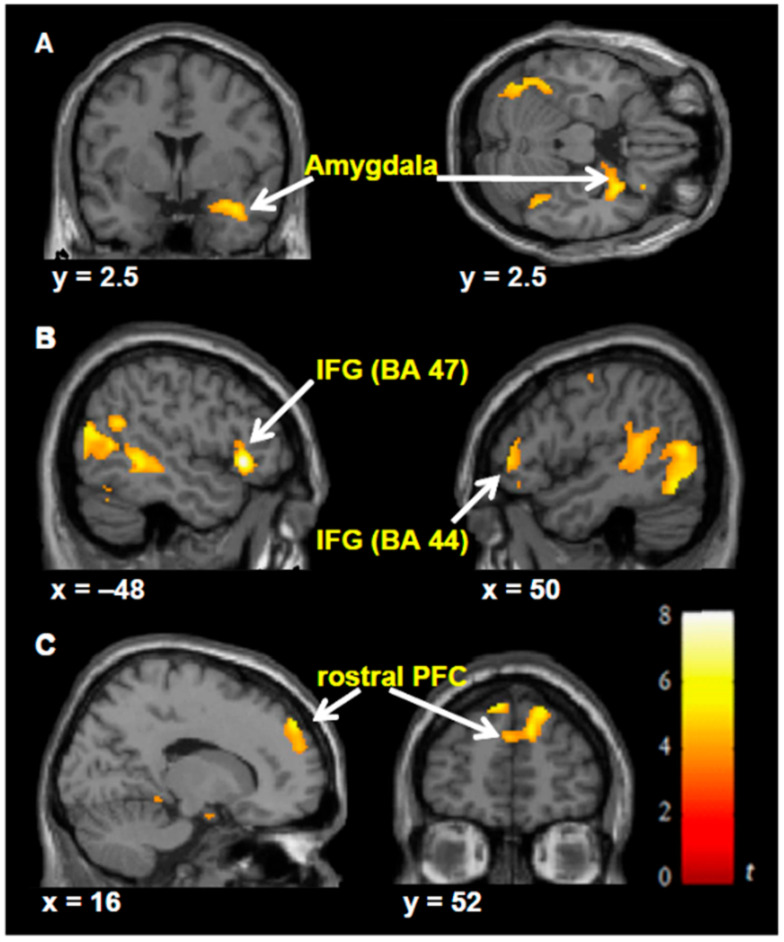
Significant hemodynamic activity during observation of avatar in pain compared with not in pain. Observing the avatar in pain compared with no pain-evoked differences in brain activation in the right amygdala and periamygdala areas (**A**), Inferior frontal gyrus (IFG)-Brodmann’s area (BA 47, BA44) (**B**), and bilateral rostral prefrontal cortex (rPFC) (**C**). Images are superimposed on the coronal and sagittal sections of the single subject structural Montreal Neurological Institute (MNI), template. All rights reserved, with permission from Frontiers. Reference [42].

Cheetham et al. [42] partially replicated Milgram’s obedience paradigm in VR in which, a virtual female was pained by the participant while observing her suffering. The event-related fMRI data was acquired and studied during the observation of pain, which showed a distinct pattern of brain response with pain-related behaviors together with an aversive state of personal distress (Figure 5).

### 3.4. Sense of Presence

Sense of presence in virtual environments is defined by an experience that provides a perception of being in a different environment than the actual. Studying the neural correlates of the sense of presence is of particular importance to researchers. Neural correlates of spatial presence can be studied with a combination of VR and EEG setup as performed by Baumgartner et al. [43], with virtual roller coaster scenarios. Besides, they used psychophysiological and psychometric measures to understand the responses. The results showed that in adolescents, activation increases in prefrontal areas, which are involved in the control of executive functions. However, in children, a decrease in activity was observed, which implies that frontal cortex functions are not fully developed in them.

Slobounov et al. [44] compared EEG correlates of a fully immersive 3D scene of spatial navigation with a 2D version and found that 3D needs a higher subjective sense of presence with the allocation of more brain and sensory resources.

Kober and Neuper [45] assessed the presence of participants in VR using auditory event-related potentials of the EEG in a virtual city. They observed that decreasing late negative slow wave amplitudes corresponded to increased presence experience. It was associated with central stimulus processing and allocation of attentional resources, and frontal negative slow waves thus were able to accurately predict the presence.

### 3.5. Spatial Navigation and Memory

Neural correlates of spatial navigation and memory-based experiments can be performed by combining VR with neuroimaging studies. Kober et al. [13] analyzed theta oscillations with EEG recording during spatial navigation in virtual environments, with a virtual maze. They observed absolute band power and event-related desynchronization/synchronization to see sex differences and reported that in the processing of navigational aid as landmarks, females showed stronger theta oscillations than men, which can indicate that females may have stronger sensorimotor integration. Similarly, Clemente et al. [46] studied the navigation control with VR-based stimulations, for which they observed the EEG activity of the right Insula for the theta band oscillation. They performed a free navigation experiment in a virtual environment, which was compared with video and photographs. They observed a significant difference between the navigation and video conditions in the right Insula activity for theta band oscillation, which is associated with stimulus attention and self-awareness processes.

Slobounov et al. [47] worked with 15 athletes having a mild traumatic brain injury, with a paradigm of the navigable virtual corridor. The BOLD response showed that concussed subjects have larger cluster sizes during encoding at the parietal cortex, right hippocampus, and the right dorsolateral prefrontal cortex. Hung et al. [48] observed a compromised speed control during basic driving conditions in cerebellum-damaged drivers. They used fMRI with VR-based driving simulator and studied motor-speed coordination in basic driving maneuvers. They observed a significant negative correlation between the mean cerebellar activation and the average time to complete right turns, which shows compromised motor coordination.

**Figure 6:**
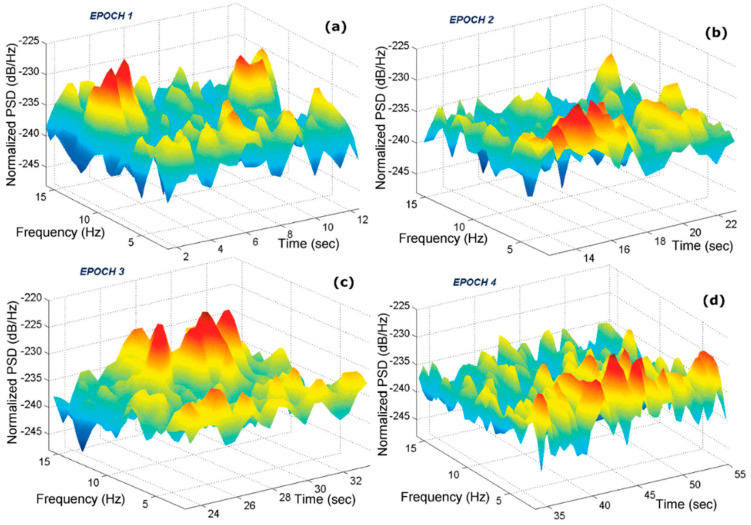
Time–frequency analysis of the magnetoencephalography (MEG) from a representative subject. (**a**–**d**) The graphs show the data parsed into epochs. Notice that Epochs 1 and 3 are dominated by alpha (**a**,**c**), whereas Epochs 2 and 4, corresponding to periods of navigation, are dominated by theta (**b**,**d**). All rights reserved, with permission from the Massachusetts Institute of Technology. Reference [50].

Ehinger et al. [49] observed a modulated alpha activity in spatial navigation due to kinesthetic and vestibular information, which was provided through a VR system in which the subject was traversing on a triangular path. Through EEG data, they observed an alpha suppression during turning movement, in parietal, occipital, and temporal clusters. This shows an increased requirement of visuo-attentional processing, thus exposing the neural correlates of spatial navigation. Araujo et al. [50] used MEG with VR-based navigation in town and studied the alpha and theta band activity. During navigation tasks, theta power was observed as strongest but, during control, no consistent increase in theta power was observed (as shown in Figure 6), thus showing a link between navigation and theta activity.

**Figure 7:**
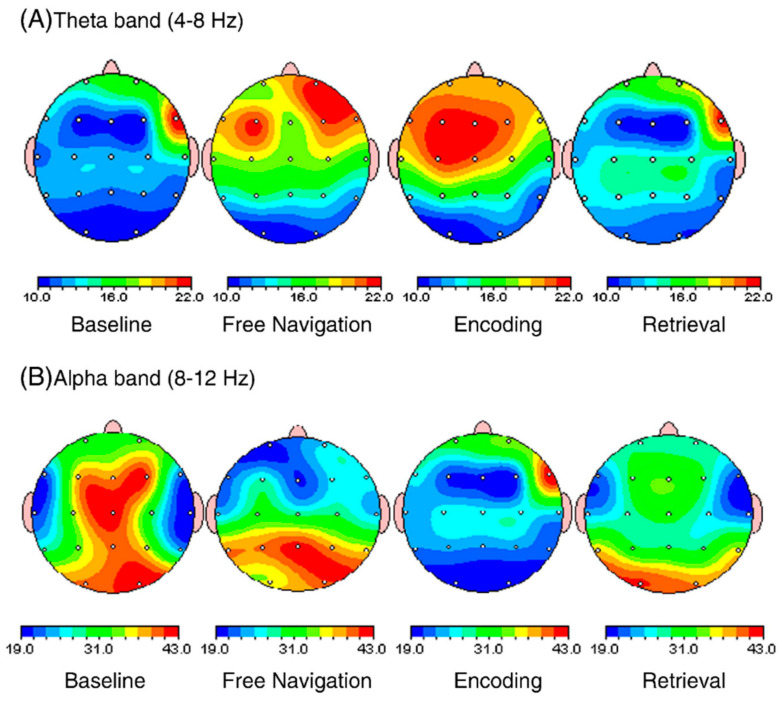
Brain maps showing topographical distribution of theta and alpha power during free navigation, baseline, encoding, and retrieval of visual–spatial working memory task. (**A**) brain map showing increase in theta power in right frontal and frontal central region during encoding and free navigation as compared to retrieval. When encoding is compared with baseline, the increase is again significant in right frontal brain region. Another point of interest is higher theta power at frontal central areas during free navigation as compared to retrieval. (**B**) brain map showing a significant increase in alpha power in all the regions during baseline in comparison to encoding and retrieval. When comparing encoding and retrieval, alpha power is slightly higher during retrieval at right parietal, left central and left, and right occipital brain regions. When comparing retrieval and free navigation, alpha power is reduced at occipital areas. Overall, both encoding and retrieval attenuate alpha power at all regions of interest (ROI). Adapted from brain research Jaiswal, N., Ray, W., and Slobounov, S. (2010). Encoding of visual–spatial information in working memory requires more cerebral efforts than retrieval: Evidence from an EEG and virtual reality study, 1347, 80–89. Copyright © 2020 Elsevier Ltd. All rights reserved, with permission from Elsevier. Reference [51].

The approach has applicability in performing memory-based experiments also. Jaiswal et al. [51] used a VR setup and observed modulation in EEG signals to compare encoding with the retrieval of visual-spatial information in working memory. The VR system provided a virtual corridor, with a sense of presence to the subjects, in which, they were able to freely navigate using their right thumb in multiple directions. They observed that theta activity increased during encoding when compared with the retrieval process (as shown in Figure 7). Further, the alpha activity was found to be significantly higher for retrieval than in the encoding process. This implied that more cerebral effort is required in the encoding of working memory than in retrieval.

### 3.6. BCI and Feedback Control

**Figure 8:**
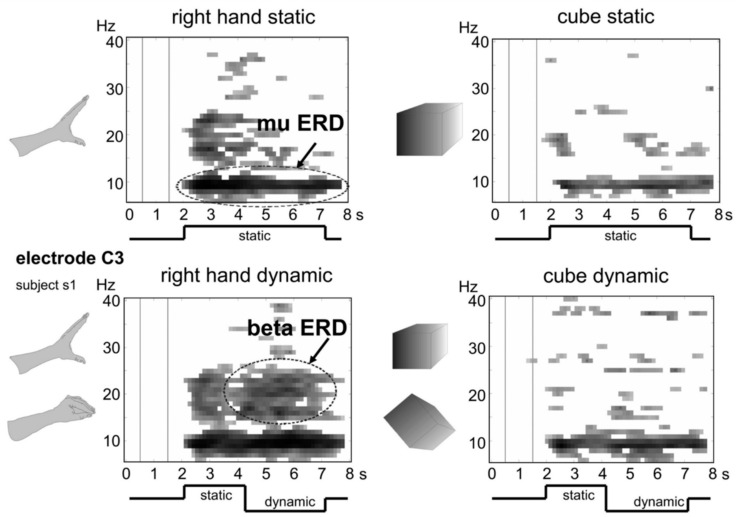
ERD/ERS time-frequency maps (6–40 Hz, 0–8 s) from bipolar recording over left cortical hand representation area of subject S1. A significant ERD in the alpha band in all four conditions is visible, along with an initial beta ERD during static and dynamic hand presentation and a further beta ERD only during observation of hand movement. All rights reserved, with permission from the Massachusetts Institute of Technology. Reference [52].

Various BCI experiments can be performed with VR devices clubbed with neuroimaging modalities, such as EEG. Pfurtscheller et al. [52] worked with a BCI system in which moving objects were presented through VR and EEG was used to study the dynamics of sensorimotor rhythms. They presented 3D moving objects (hand and cube) and noted that a stronger desynchronization of the central beta rhythm is present in moving hand, in comparison to moving cube, inferring that the type of object matters in neural processing. Moreover, it may suggest a greater involvement of motor areas in processing body parts-based objects than others. Figure 8 shows the corresponding Event-related de-synchronization/Event-related synchronization (ERD/ERS) time-frequency maps.

In BCI, feedback is an important component, and VR tools can improve the BCI-feedback presentation. Ron-Angevin and Díaz-Estrella [53] explored this function and trained subjects using the BCI system with a virtual environment. This helped to modify EEG behavior through improvement in feedback control. Similarly, Othmer et al. [54] worked for the implementation of EEG biofeedback with VR-based stimulation in subjects with neurological disorders. Zarka et al. [55] researched the time course of scalp activation during observation of human gait with EEG and human walking 3D-animation in normal, upside down, and uncoordinated conditions. They observed a decreased P120 response in the upside-down condition and a decreased N170 and P300b amplitude in the uncoordinated condition, together with decreased alpha power and theta phase locking in both conditions. The results were in agreement with the various existing point-light display studies; thus, supporting the application of VR for neurofeedback. Vourvopoulos et al. [6] utilized VR-based BCI neurofeedback for stroke patient rehabilitation, for which they used a platform that can acquire post-stroke EEG signals with function of movement of a virtual avatar arm, which allowed a subject-driven action observation neurofeedback in VR. The results showed that EEG based neurofeedback is beneficial for severe motor impairment patients.

However, various neurotechnological advancements, such as an enhanced compatibility with the full functionality of VR systems with neuroimaging modalities (such as fMRI and MEG), enhanced the degree of freedom for mobility in an integrated system; hybrid neuroimaging with VR stimulations, etc. are yet to be researched, which can provide a robust system of integrated VR environment and BCI systems.

### 3.7. Neuromarketing

Neuromarketing involves the implication of neuropsychology in marketing research to assess the neural responses from the subject for marketing stimuli [56,57]. The addition of VR technologies can give an extra edge to neuromarketing by providing a virtual environment to show marketing creativity. Burris et al. [58] contributed an article describing the applicability of an integrated VR-neuroimaging system in neuromarketing. The chapter provided an overview of various means of studying human neurological responses with VR stimulated marketing environment, through neuroimaging modalities such as EEG, MEG, and fMRI. Castellanos et al. [59] explored the neuromarketing approach in digital video advertising with EEG measurements. It consisted of interactive videos shown on the 360° screen followed by a questionnaire. The study evaluated the effect of interactivity in emotion during the viewing of the advertisements. The power spectral density (PSD) values of all frequency bands were computed for the 360° video stimulations. The frontal asymmetry index and difference score to summarize relative activity at homologous right and left sites, were calculated, and difference scores were averaged. A larger index of frontal asymmetry was observed for the 360° Group in comparison to the non 360° group. Thus, immersive, and interactive features of a 360° advertisement were found to enhance the emotional responses of valence.

However, the application of VR systems in neuromarketing needs further research to understand the vibrant perspective of marketing approaches and associated neural correlates.

### 3.8. Other Fields

Dynamic stimulations with VR can mimic a haptic environment and can be utilized to study various sensations, such as motion sickness. Lin et al. [60] worked with a dynamic VR environment consisting of a 3D surrounding VR scene and a motion platform. Sickness levels were studied using EEG signals with independent component analysis and it was observed that in the parietal and motor areas, power of 8–10 Hz frequency band increased with motion sickness-related events. This helped to assess the neural correlates of motion sickness.

VR can be used to study depression and its neural correlates. Depression is sometimes envisaged to be a result of the dysfunctional hippocampus, and this can be studied using MEG scans with VR navigation tasks, as experimented by Cornwell et al. [61]. The subjects were asked to navigate a virtual Morris water maze to find a hidden platform and theta oscillations (4–8 Hz) were mapped across the brain. Dysfunction of the right anterior hippocampus and para- hippocampal cortices were indicated as the reason for depression due to observation of the comparative differences in theta activity in right medial temporal cortices.

In addition, researchers have been trying to induce a hypnotic effect through VR systems. White et al. [62] studied the effect of hypnosis induced by a VR hypnosis induction system, by investigating EEG-based coherence and power spectra changes. The VR system consisted of a spoken hypnotic induction system with instructions to relax and was presented with scenes and sounds in the background. A decrease in incoherence was observed in the high susceptibility group while an increase in coherence was noted in the low susceptibility group, between medial frontal and lateral left prefrontal sites.

These discussed approaches and applicability in multidimensional fields show the efficacy of VR based research together with deciphering the neuronal response. Table 1 provides an overview of the discussed research, showing the synergistic relation and applicability of VR systems in neuroimaging research.

## 4. Limitations and Future Perspective

There is a significant and considerable difference between VR and actual reality, which is due to the factors of perception and human sense. The present VR devices lack intensive immerse experience up to the level of actual reality, and research studies and technology firms lack a quantifiable dataset to describe and measure this difference. Moreover, the complete and integrated virtual experience is lacking a perfect association of environmental and stimulation factors, including, audiovisual dynamics, haptics, and other sensory input. Furthermore, the neuronal response from the brain as sensory feedback is not yet completely integrated with VR-based devices, which can provide a complete solution to various real-life scenarios. In addition, research is lacking on studying and quantifying how much reality we have achieved in these VR devices. There is a requirement to develop new approaches and quantify the perception of reality in VR devices, and to develop tools for quantification of reality in VR devices based on neural correlates and responses. Approaches that can assist in integrating neuronal signals for real-time feedback, and control and enhance the VR experience are also required. These technical issues are getting the attention of researchers and are expected to be developed and advanced in the near future.

## 5. Conclusions

This article presented an overview of the synergistic integration of VR-based systems with neuroimaging modalities and explained their integration approach. The current evidence from research shows that VR-based environment creation can be utilized to perform various experiments from different fields of cognitive sciences, engineering, and biomedical engineering, and sciences. It has immense applicability and efficacy and can provide an insight into the functioning of the brain in response to dynamic stimuli. An overview of various experiments having applications in neurorehabilitation and therapy, neuromodulation, cognition, perception, navigation, memory encoding and retrieval, BCI, and in emerging fields, such as neuromarketing, is provided in this review, which will help researchers to explore further possibilities and do technological advancements in this multidisciplinary field. Furthermore, there is a need to work on the discussed limitations and perform potential research in other fields, such as social interaction and neurotechnology, together with the development of a fully functional industrial applicability.

## Figures and Tables

**Table 1:** An overview of synergistic relation and applicability of VR systems in neuroimaging research.

Serial. Number	Neuroimaging Technique	VR Stimulation System	Neural Correlates/Area of Interest	References
**1.** (a)	**EEG**	VR system with a series of scenes associated with virtual alcohol cues, relaxation, and aversive stimulation.	Study of Alpha activity in the frontal region.	[25]
(b)		Robotic-assisted gait training device together with VR scenes for neurorehabilitation.	Entrainment of several brain areas and stronger cortical activations due to VR feedback.	[28]
(c)		VR-based prosthetics training for stroke patients.	Activation of primary and secondary motor areas through VR-based exercise, working as neuromotor rehabilitation.	[29,30]
(d)		VR-based attention enhancement system, integrated with EEG biofeedback.	Treatment for Attention disorder, using Beta wave as feedback control and showed significant results in attention enhancement.	[37]
(e)		VR device, which can perform traffic-light motion simulation to study driver cognitive response, by kinetic audiovisual stimuli delivering.	ERP signals processed with a self-constructing neural fuzzy inference network, which recognized different brain potentials corresponding to red/green/yellow traffic events.	[39]
(f)		Varying motion speed of a non-immersive 3D virtual stimulus.	Theta band power in the dorsolateral prefrontal cortex.	[40]
(g)		VR system for inducing the visual perception of manipulable objects for motor coding of visual objects in peripersonal space.	Centro-parietal region was studied while perceptually judging the intrinsic or extrinsic properties of visual objects.	[14]
(h)		Virtual roller coaster scenarios to study the neural correlates of sense of presence.	Activation increased in prefrontal areas that are involved in the control of executive functions.	[43]
(i)		Spatial navigation and memory-based experiments with a virtual maze.	Theta oscillations study during spatial navigation. Observing absolute band power and event-related synchronization.	[13]
(j)		Free navigation experiment in a virtual environment, which was compared with video and photographs.	EEG activity of the right Insula for the theta band oscillation.	[46]
(k)		VR system in which the subject was traversing on a triangular path.	Modulated alpha activity in spatial navigation due to kinesthetic and vestibular information. Alpha suppression during turning movement, in parietal, occipital, and temporal clusters	[49]
(l)		Brain computer interface (BCI) system in which moving objects were presented through VR.	Dynamics of sensorimotor rhythms.	[52]
(m)		VR-based BCI neurofeedback system for stroke patient rehabilitation, with the function of the movement of a virtual avatar arm.	EEG based neurofeedback beneficial for severe motor impairment patients.	[6]
(n)		Digital video advertising with EEG measurements 360-degree screen for neuromarketing study.	Study the effect of interactivity in emotion during the viewing of the advertisements. The power spectral density (PSD) values of all frequency bands were computed with an index of frontal asymmetry.	[59]
**2.** (a)	**fMRI**	Motor functions in neurodegenerative diseases, such as freezing behavior, provoked through VR-based stimulation method.	BOLD patterns observed during walking and episodes of freezing.	[27]
(b)		VR-based prosthetics training for stroke patients.	Activation of primary and secondary motor areas through VR-based exercise, working as neuromotor rehabilitation.	[32]
(c)		VR-based rehabilitation gaming system for stroke patients.	Object processing, attention, and mirror neuron system involvement. Virtual target catching can activate frontal, parietal, temporal, cingulate, and cerebellar regions.	[33]
(d)		Virtual reality enhanced treadmill.	BOLD response for ankle dorsiflexion activity showed increased activation in the primary sensorimotor cortex of the lesioned hemisphere and in supplementary motor areas.	[34]
(e)		MRI compatible VR-based setup that can trigger illusory sensations with visual and tactile stimulations.	BOLD response with illusory ownership experiences for a virtual limb. The bilateral activity of ventral premotor cortex, secondary somatosensory cortex, extrastriate visual cortex, as well as right-hemispheric activation of the cerebellum.	[35]
(f)		Milgram’s obedience paradigm in VR.	Event-related BOLD response during the observation of pain.	[42]
(g)		Navigable virtual corridor for mild traumatic brain injury patients	BOLD response showing that concussed subjects have larger cluster sizes during encoding at the parietal cortex, right hippocampus and, right dorsolateral prefrontal cortex.	[47]
(h)		VR-based driving simulator to study motor-speed coordination in basic driving maneuvers.	Mean cerebellar activation and the average time to complete right turns, which shows compromised motor coordination in patients.	[48]
**3.** (a)	**MEG**	Virtual system to study neural correlates of auditory distance perception.	MEG signals as variation in amplitude over both supratemporal planes. Hemispheric lateralization was indicated by enhanced pre-attentive responses over the right temporal lobe.	[41]
(b)		VR-based navigation in a virtual town.	Alpha and theta band activity. Showed a link between navigation and theta activity.	[50]
(c)		Virtual Morris water maze to find a hidden platform as a virtual reality navigation task.	Theta oscillations mapping across the brain. Dysfunction of the right anterior hippocampus and para- hippocampal cortices were indicated as the reason for depression due to observation of the comparative difference in theta activity in right medial temporal cortices.	[61]

## Data Availability

No new data were created or analyzed in this study. Data sharing is not applicable to this article.

## References

[1] Geoffrey W.G. (2014). Using virtual reality to augment perception, enhance sensorimotor adaptation, and change our minds. Front. Syst. Neurosci..

[2] Pandarinathan G., Mishra S., Nedumaran A.M., Padmanabhan P., Gulyás B. (2018). The potential of cognitive neuroimaging: A way forward to the mind-machine interface. J. Imaging.

[3] Lécuyer A., Lotte F., Reilly R.B., College T. (2008). Brain-Computer Interfaces, Virtual Reality, and Videogames. Computer.

[4] Padmanabhan P., Nedumaran A.M., Mishra S., Pandarinathan G., Archunan G., Gulyás B. (2017). The Advents of Hybrid Imaging Modalities: A New Era in Neuroimaging Applications. Adv. Biosyst..

[5] Luu T.P., Nakagome S., He Y., Contreras-Vidal J.L. (2017). Real-time EEG-based brain-computer interface to a virtual avatar enhances cortical involvement in human treadmill walking. Sci. Rep..

[6] Vourvopoulos A., Pardo O.M., Lefebvre S., Neureither M., Saldana D., Jahng E., Liew S.-L. (2019). Effects of a brain-computer interface with virtual reality (VR) neurofeedback: A pilot study in chronic stroke patients. Front. Hum. Neurosci..

[7] Tremmel C., Herff C., Sato T., Rechowicz K., Yamani Y., Krusienski D.J. (2019). Estimating cognitive workload in an interactive virtual reality environment using EEG. Front. Hum. Neurosci..

[8] Piccione J., Collett J., De Foe A. (2019). Virtual skills training: The role of presence and agency. Heliyon.

[9] David N., Skoruppa S., Gulberti A., Schultz J., Engel A.K. (2016). The sense of agency is more sensitive to manipulations of outcome than movement-related feedback irrespective of sensory modality. PLoS ONE.

[10] Kilteni K., Grau-Sánchez J., Veciana De Las Heras M., Rodríguez-Fornells A., Slater M. (2016). Decreased corticospinal excitability after the illusion of missing part of the arm. Front. Hum. Neurosci..

[11] Cebolla A.M., Petieau M., Cevallos C., Leroy A., Dan B., Cheron G. (2015). Long-lasting cortical reorganization as the result of motor imagery of throwing a ball in a virtual tennis court. Front. Psychol..

[12] Sangani S., Lamontagne A., Fung J. (2015). Cortical mechanisms underlying sensorimotor enhancement promoted by walking with haptic inputs in a virtual environment. Progress in Brain Research.

[13] Kober S.E., Neuper C. (2011). Sex differences in human EEG theta oscillations during spatial navigation in virtual reality. Int. J. Psychophysiol..

[14] Wamain Y., Gabrielli F., Coello Y. (2016). EEG μ rhythm in virtual reality reveals that motor coding of visual objects in peripersonal space is task dependent. Cortex.

[15] Qin Y., Bulbul T. (2020). Towards an EEG Based Mental Workload Evaluation Method for Construction Workers’ HMD AR Use. Proceedings of the Construction Research Congress 2020: Computer Applications.

[16] Rho G., Callara A.L., Condino S., Ghiasi S., Nardelli M., Carbone M., Ferrari V., Greco A., Scilingo E.P. A preliminary quantitative EEG study on Augmented Reality Guidance of Manual Tasks. Proceedings of the 2020 IEEE International Symposium on Medical Measurements and Applications (MeMeA).

[17] Ke Y., Liu P., An X., Song X., Ming D. (2020). An online SSVEP-BCI system in an optical see-through augmented reality environment. J. Neural Eng..

[18] Hari R., Salmelin R. (1997). Human cortical oscillations: A neuromagnetic view through the skull. Trends Neurosci..

[19] Leroy A., Cevallos C., Cebolla A.-M., Caharel S., Dan B., Cheron G. (2017). Short-term EEG dynamics and neural generators evoked by navigational images. PLoS ONE.

[20] Cebolla A.-M., Palmero-Soler E., Leroy A., Cheron G. (2017). EEG spectral generators involved in motor imagery: A swLORETA Study. Front. Psychol..

[21] Roberts G., Holmes N., Alexander N., Boto E., Leggett J., Hill R.M., Shah V., Rea M., Vaughan R., Maguire E.A. (2019). Towards OPM-MEG in a virtual reality environment. Neuroimage.

[22] Adamovich S.V., August K., Merians A., Tunik E. (2009). A virtual reality-based system integrated with fmri to study neural mechanisms of action observation-execution: A proof of concept study. Restor. Neurol. Neurosci..

[23] Macaluso E., Ogawa A. (2018). Visuo-spatial orienting during active exploratory behavior: Processing of task-related and stimulus-related signals. Cortex.

[24] Ku J., Mraz R., Baker N., Zakzanis K.K., Lee J.H., Kim I.Y., Kim S.I., Graham S.J. (2003). A data glove with tactile feedback for FMRI of virtual reality experiments. Cyberpsychol. Behav..

[25] Lee S.H., Han D.H., Oh S., Lyoo I.K., Lee Y.S., Renshaw P.F., Lukas S.E. (2009). Quantitative electroencephalographic (qEEG) correlates of craving during virtual reality therapy in alcohol-dependent patients. Pharmacol. Biochem. Behav..

[26] Moon J., Lee J.H. (2009). Cue exposure treatment in a virtual environment to reduce nicotine craving: A functional MRI study. Cyberpsychol. Behav..

[27] Shine J.M., Ward P.B., Naismith S.L., Pearson M., Lewis S.J.G. (2011). Utilising functional MRI (fMRI) to explore the freezing phenomenon in Parkinson’s disease. J. Clin. Neurosci..

[28] Calabrò R.S., Naro A., Russo M., Leo A., De Luca R., Balletta T., Buda A., La Rosa G., Bramanti A., Bramanti P. (2017). The role of virtual reality in improving motor performance as revealed by EEG: A randomized clinical trial. J. Neuroeng. Rehabil..

[29] August K., Lewis J.A., Chandar G., Merians A., Biswal B., Adamovich S. fMRI analysis of neural mechanisms underlying rehabilitation in virtual reality: Activating secondary motor areas. Proceedings of the 2006 International Conference of the IEEE Engineering in Medicine and Biology Society.

[30] Steinisch M., Tana M.G., Comani S. (2013). A Post-Stroke Rehabilitation System Integrating Robotics, VR and High-Resolution EEG Imaging. IEEE Trans. Neural Syst. Rehabil. Eng..

[31] Gonçalves M.G., Piva M.F.L., Marques C.L.S., da Costa R.D.M., Bazan R., Luvizutto G.J., Betting L.E.G.G. (2018). Effects of virtual reality therapy on upper limb function after stroke and the role of neuroimaging as a predictor of a better response. Arq. Neuropsiquiatr..

[32] Merians A.S., Tunik E., Adamovich S.V. (2009). Virtual reality to maximize function for hand and arm rehabilitation: Exploration of neural mechanisms. Stud. Health Technol. Inform..

[33] Prochnow D., Bermudez I., Badia S., Schmidt J., Duff A., Brunheim S., Kleiser R. (2013). A functional magnetic resonance imaging study of visuomotor processing in a virtual reality-based paradigm: Rehabilitation gaming system. Eur. J. Neurosci..

[34] Xiao X., Lin Q., Lo W.-L., Mao Y.-R., Shi X., Cates R.S., Zhou S.-F., Huang D.-F., Li L. (2017). Cerebral reorganization in subacute stroke survivors after virtual reality-based training: A preliminary study. Behav. Neurol..

[35] Bach F., Çakmak H., Maaß H., Bekrater-Bodmann R., Foell J., Diers M., Trojan J., Fuchs X., Flor H. (2012). Illusory hand ownership induced by an MRI compatible immersive virtual reality device. Biomed. Tech..

[36] Menon S., Brantner G., Aholt C., Kay K., Khatib O. Haptic fMRI: Combining functional neuroimaging with haptics for studying the brain’s motor control representation. Proceedings of the 35th Annual International Conference of the IEEE Engineering in Medicine and Biology Society (EMBC).

[37] Cho B.H., Lee J.M., Ku J.H., Jang D.P., Kim J.S., Kim I.Y., Lee J.H., Kim S.I. Attention Enhancement System Using Virtual Reality and EEG Biofeedback. Proceedings of the Virtual Reality Annual International Symposium.

[38] Choo A., May A. Virtual Mindfulness Meditation: Virtual Reality and Electroencephalography for Health Gamification. Proceedings of the 2014 IEEE Games Media Entertainement Conference.

[39] Lin C.T., Chung I.F., Ko L.W., Chen Y.C., Liang S.F., Duann J.R. (2007). EEG-based assessment of driver cognitive responses in a dynamic virtual-reality driving environment. IEEE Trans. Biomed. Eng..

[40] Martins e Silva D.C., Marinho V., Teixeira S., Teles G., Marques J., Escórcio A., Fernandes T., Freitas A.C., Nunes M., Ayres M. (2020). Non-immersive 3D virtual stimulus alter the time production task performance and increase the EEG theta power in dorsolateral prefrontal cortex. Int. J. Neurosci..

[41] Mathiak K., Hertrich I., Kincses W.E., Riecker A., Lutzenberger W., Ackermann H. (2003). The right supratemporal plane hears the distance of objects: Neuromagnetic correlates of virtual reality. Neuroreport.

[42] Cheetham M., Pedroni A.F., Antley A., Slater M., Jäncke L. (2009). Virtual milgram: Empathic concern or personal distress? Evidence from functional MRI and dispositional measures. Front. Hum. Neurosci..

[43] Baumgartner T., Valko L., Esslen M., Jäncke L. (2006). Neural correlate of spatial presence in an arousing and noninteractive virtual reality: An EEG and psychophysiology study. Cyberpsychol. Behav..

[44] Slobounov S.M., Ray W., Johnson B., Slobounov E., Newell K.M. (2015). Modulation of cortical activity in 2D versus 3D virtual reality environments: An EEG study. Int. J. Psychophysiol..

[45] Kober S.E., Neuper C. (2012). Using auditory event-related EEG potentials to assess presence in virtual reality. Int. J. Hum. Comput. Stud..

[46] Clemente M., Rodríguez A., Rey B., Alcañiz M. (2014). Assessment of the influence of navigation control and screen size on the sense of presence in virtual reality using EEG. Expert Syst. Appl..

[47] Slobounov S.M., Zhang K., Pennell D., Ray W., Johnson B., Sebastianelli W. (2010). Functional abnormalities in normally appearing athletes following mild traumatic brain injury: A functional MRI study. Exp. Brain Res..

[48] Hung Y., Vetivelu A., Hird M.A., Yan M., Tam F., Graham S.J., Cusimano M., Schweizer T.A. (2014). Using fMRI virtual-reality technology to predict driving ability after brain damage: A preliminary report. Neurosci. Lett..

[49] Ehinger B.V., Fischer P., Gert A.L., Kaufhold L., Weber F., Pipa G., König P. (2014). Kinesthetic and vestibular information modulate alpha activity during spatial navigation: A mobile EEG study. Front. Hum. Neurosci..

[50] De Araújo D.B., Baffa O., Wakai R.T. (2002). Theta oscillations and human navigation: A magnetoencephalography study. J. Cogn. Neurosci..

[51] Jaiswal N., Ray W., Slobounov S. (2010). Encoding of visual-spatial information in working memory requires more cerebral efforts than retrieval: Evidence from an EEG and virtual reality study. Brain Res..

[52] Pfurtscheller G., Scherer R., Leeb R., Keinrath C., Neuper C., Lee F., Bischof H. (2007). Viewing moving objects in virtual reality can change the dynamics of sensorimotor EEG rhythms. Presence Teleoper. Virtual Environ..

[53] Ron-Angevin R., Díaz-Estrella A. (2009). Brain-computer interface: Changes in performance using virtual reality techniques. Neurosci. Lett..

[54] Othmer S., Kaiser D. (2000). Implementation of virtual reality in EEG biofeedback. Cyberpsychol. Behav..

[55] Zarka D., Cevallos C., Petieau M., Hoellinger T., Dan B., Cheron G. (2014). Neural rhythmic symphony of human walking observation: Upside-down and Uncoordinated condition on cortical theta, alpha, beta and gamma oscillations. Front. Syst. Neurosci..

[56] Ariely D., Berns G.S. (2010). Neuromarketing: The hope and hype of neuroimaging in business. Nat. Rev. Neurosci..

[57] Mishra S. (2019). Neuromarketing: Neural Explanations for Consumer Behaviours. Biomed. J. Sci. Tech. Res..

[58] Burris H.R., Sheikh S.A. (2010). Virtual reality and neuroimaging technologies: Synergistic approaches in neuromarketing. Virtual Technologies for Business and Industrial Applications: Innovative and Synergistic Approaches.

[59] Castellanos M.C., Ausin J.M., Guixeres J., Bigné E. (2018). Emotion in a 360-Degree vs. Traditional Format Through EDA, EEG and Facial Expressions. Adv. Advert. Res..

[60] Lin C.T., Chuang S.W., Chen Y.C., Ko L.W., Liang S.F., Jung T.P. EEG effects of motion sickness induced in a dynamic virtual reality environment. Proceedings of the 2007 29th Annual International Conference of the IEEE Engineering in Medicine and Biology Society.

[61] Cornwell B.R., Salvadore G., Colon-Rosario V., Latov D.R., Holroyd T., Carver F.W., Coppola R., Manji H.K., Zarate C.A., Grillon C. (2010). Abnormal hippocampal functioning and impaired spatial navigation in depressed individuals: Evidence from whole-head magnetoencephalography. Am. J. Psychiatry.

[62] White D., Ciorciari J., Carbis C., Liley D. (2009). EEG correlates of virtual reality hypnosis. Int. J. Clin. Exp. Hypn..

